# Deep Learning and Drug Discovery for Healthy Aging

**DOI:** 10.1021/acscentsci.3c01212

**Published:** 2023-10-17

**Authors:** Peter Wang, Dean Ho

**Affiliations:** † Institute for Digital Medicine (WisDM), Yong Loo Lin School of Medicine, National University of Singapore, Singapore 119077; ‡ The N.1 Institute for Health (N.1), National University of Singapore, Singapore 119077; § Department of Biomedical Engineering, College of Design and Engineering, National University of Singapore, Singapore 119077; ∥ Department of Pharmacology, Yong Loo Lin School of Medicine, National University of Singapore, Singapore 119077; ⊥ Singapore’s Health District @ Queenstown, Yong Loo Lin School of Medicine, National University of Singapore, Singapore 119077

Osteoporosis is a globally prevalent disorder that leads to substantial increases in mortality, morbidity, and healthcare costs.^1^ In the United States alone, approximately 1.5 million osteoporosis-driven fractures have led to hundreds of thousands of hospitalizations annually. In parts of the world characterized by rapidly aging populations and superaged societies, as high as 30% of women aged 40 and up are estimated to be affected by osteoporosis.^2^ Given the rising life expectancy of countries in Asia Pacific, osteoporotic and other bone disorders merit major attention in order to address healthcare costs and patient mortality and morbidity. One key strategy to address these issues is the rapid development of safe and effective therapies.

Toward the development of potential interventions for bone diseases, a long-standing barrier in bone tissue engineering—from fundamental research to patient-level applications—has been the identification of clinically actionable agents to maintain the stemness of bone marrow mesenchymal stem cells (BMMSCs). Due to the role of BMMSCs in regeneration and homeostasis of bone tissue, preserving stemness has been long sought after in the development of effective treatments for bone diseases. Prior approaches have involved hormone or bone resorption treatment.^3,4^ However, these strategies do not address the core issue of stemness. Other approaches that address stemness included cytokine therapy and gene editing.^5,6^ Beyond rapidly developing bone disease treatment options that are safe and effective, their accessibility and affordability remain key attributes that also need to be considered.

In this issue of *ACS Central Science*, Liu, Li, Xie, and co-workers have harnessed a deep learning (DL) platform to rapidly pinpoint a potential drug candidate for osteoporosis.^7^ Specifically, this study prioritized dihydroartemisinin (DHA), a broadly available natural compound to maintain desired differentiation and self-renewal properties *in vitro* and bone density and architectural integrity *in vivo.* The DL platform used in this study, termed the deep learning efficacy prediction system (DLEPS), functions by harnessing data that correlates drug interactions with the transcriptome. This information can then be used for additional assessment against differentially expressed genes (DEGs) to produce an efficacy score to prioritize drug testing. To provide further insight into the development of this efficacy score, this study used RNA sequencing to assess which DEGs, measured from day 1 postnatal to 6–8-week-old adult mice, were downregulated during the aging process. The DLEPS produced a ranking from a 12,328-gene set to predict DEG reversal in order to preserve stemness. From this data set, a bone score was generated by integrating DEG up-/downregulation analysis in order to prioritize compounds for further evaluation.

In this study, DLEPS prediction identified DHA as a promising agent for further evaluation. The administration of 0.1 μm DHA to human BMMSCs (hBMMSCs) increased OCT4 and SOX2 expression. Of note, adding DHA to the culture media sustained increased OCT4 and SOX2 expression for five passages. This was accompanied by Ki67 staining, which further demonstrated improved hBMMSC proliferation when treated with DHA. In addition, to assess hBMMSC capacity for osteogenic differentiation, DHA administration resulted in enhanced mineralization (alizarin red staining/ARS) as well as alkaline phosphatase (ALP) activity compared to controls. To demonstrate DHA-driven reduction of adipogenic differentiation capacity, Oil red O-labeling revealed a reduced lipid droplet presence compared to controls.

To acquire deeper insights into the mechanistic basis for DHA-enhanced stemness, the team further examined the histone deacetylase (HDAC) and histone acetyltransferase (HAT) enzymes, as the levels of these enzymes were substantially differentiated from nonosteoporotic mice. Given the previously reported role of compounds similar to DHA in H3K9 (Histone 3 Lysine 9) upregulation of acetylation in other cell types, this study then evaluated the role of DHA specifically in regulating the genes that drive stemness in a preclinical setting.^8^ Among a series of HAT (*GCN5*, *P200*, *PCAF*) and HDAC (*SIRT6*, *HDAC1*, *HDAC2*, and *HDAC8*) enzymes, *GCN5* expression increased 1.5× following DHA administration alongside increased H3K9 acetylation in osteoporotic BMMSCs, which matched *in vitro* findings. Furthermore, knockdown of *GCN5* after sustained DHA administration (Passage 8) reduced the expression of stemness markers and capacity for osteogenesis, and DHA administration in these cells did not increase stemness marker expression.

To further increase the localization of DHA delivery to the bone, the team utilized mesoporous silica nanoparticles (MSNs) conjugated to alendronates (ALNs), which target bone tissue, to form MSN-ALN vehicles. When loaded with DHA, the MSN-ALN vehicles markedly improved bone architecture and retention of bone mass in osteoporotic mice compared to the sham cohort. Of note, since ALNs have been previously shown to mitigate osteoclast functionality, it is believed that the integration of MSN-ALN vehicles with DHA collectively even further impeded osteoclast activity. Collectively, the MSN-ALN vehicles loaded with DHA also substantially enhanced osteoblast functionality over unmodified MSN-ALN, demonstrating a promising path forward for the continued evaluation of DHA-loaded nanoparticles.

**Figure 1:**
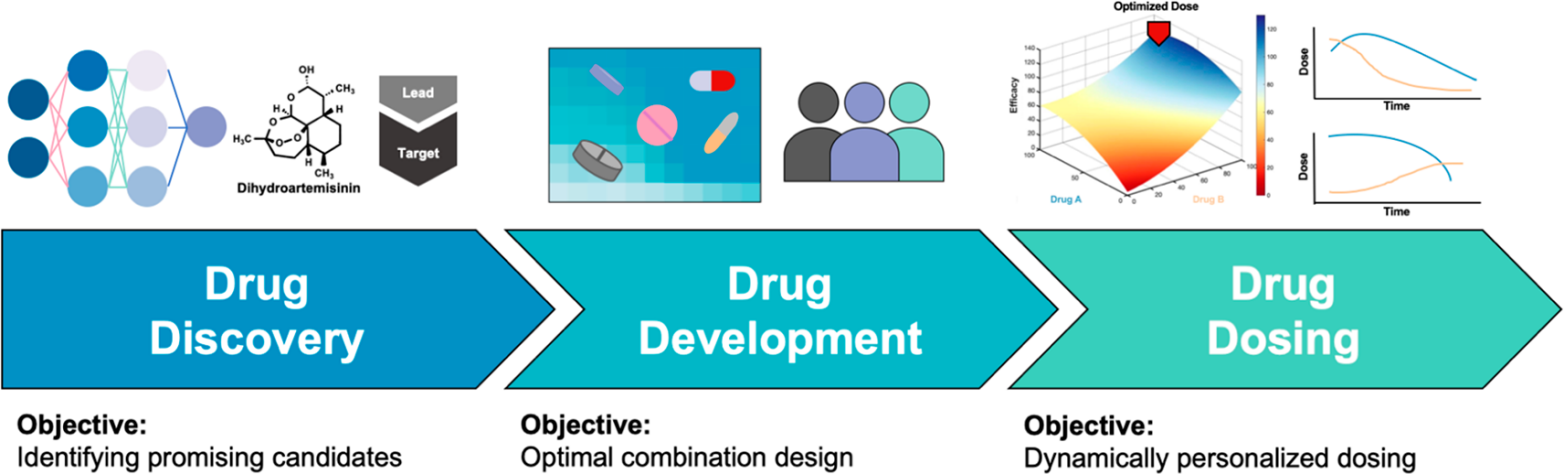
Segments of drug development workflow. Approaches like DL have opened a gateway to discover substantially more potential drug candidates (e.g., DHA). Given these DL-identified drug candidates, designing effective combination therapies requires true optimization in the large parameter search space. Furthermore, successful combination therapies may need dynamic modulations to continuously sustain the best clinical outcomes for patients.

This work represents important validation for the role of DL in rapid drug discovery. In evaluating the broader role of artificial intelligence (AI)-driven therapy, it is important to note that drug discovery is one segment that resides within a larger drug optimization workflow that includes drug development—involving the design of drug combinations among other factors—and drug dosing. Taken as a complete workflow, these three segments can profoundly impact the clinical actionability and sustained response of patients to single agent or combination regimens (Figure 1).^9^

Expanding the use of DL within the larger context of AI to rapidly discover and recommend compounds for further evaluation has opened a gateway to a wider array of potential drug candidates (Figure 1).^10^ To properly steward these promising candidates forward, harnessing true optimization to find suitable partner therapies for combination regimens may be essential. This has been especially true for other indications such as oncology, cardiac diseases, diabetes, infectious diseases, and beyond. Natural compounds are receiving increased attention due to AI/DL-predicted applications toward disease management.^11^ As they are evaluated further, toward preclinical and potential clinical studies, it is likely that combination regimens will be needed to realize their full potential for clinically significant efficacy. While mechanism-of-action (MOA) and drug sensitivity assays have traditionally been used for combination design, the use of AI may uncover unpredictable or unforeseen interactions that can markedly increase treatment efficacy. To achieve this outcome, a large parameter space needs to be explored, as even a 10-drug set with each drug studied at 10 dose levels can lead to billions of possible permutations. This would preclude conventional, iterative experiments. Fortunately, emerging AI-based strategies can interrogate parameter spaces of this magnitude by pairing prospective experiments with established optimization methods to yield globally optimized drug combinations. Examples include recent work to address SARS-CoV-2 drug development, blood cancers in patients, and antimicrobial resistance.^12−15^ In addition, a newly developed DL model, DrugCell, simulates the response of human cancer cells when treated with therapeutics and subsequently predicts synergistic drug combinations that may improve treatment outcomes.^16^

Following the drug development segment of the workflow, preclinical and clinical drug dosing optimization follows. Importantly, once DL-discovered drug candidates are validated and combination regimens are developed, optimized dosing can have a profound effect on the clinical efficacy of the candidates. Traditional approaches have defined dose optimization through dose escalation until a maximum tolerated dose (MTD) is reached. However, emerging strategies have shown that truly optimized dosing is a dynamic process, and treatment should evolve alongside the patient. Specifically, dose adjustments should potentially be modulated longitudinally in order to sustain the best outcomes possible for a patient. This is due to the observations that drug synergy is dose-dependent, time-dependent, and subject to individualized patient responses. In the case of the work reported by Liu, Li, Xie, and co-workers, there may be interactions between the MSN-ALN vehicles and DHA that collectively improve the antiosteoporotic outcomes. Recent work has shown that substantial dose reductions can maintain stable disease in human solid cancer treatment.^17^ In addition, evolutionary dynamics has been used to reduce total drug doses needed for human prostate cancer therapy.^18^ Given the aging-related nature of osteoporosis and other bone diseases, modulated dosing in accordance with physiological and disease changes that inevitably take place during chronological age progression may help prolong efficacy. The work of Liu, Li, Xie, and co-workers can potentially play an important role toward accessible and sustained intervention against age-related diseases to align healthspan with lifespan—so that the duration of a person’s health matches their duration of life.

Studies such as the work reported by Liu, Li, Xie, and co-workers represent a promising step in advancing AI/DL-identified compounds toward clinically relevant validation. In particular, expanding the repertoire of potential agents to address disorders that increase in prevalence with aging enhances the range of interventional strategies available for increasing healthspan alongside lifespan. These are particularly critical needs in many parts of the world today. In this context, when promising drug candidates are evaluated as part of an integrated workflow of drug discovery, development, and dosing, this brings to role of AI in the comprehensive and sustained optimization of patient treatment closer to validation.
